# Hints to Young Practitioners—No. III

**Published:** 1872-09

**Authors:** 

**Affiliations:** Savannah, Georgia


### HINTS TO YOUNG PRACTITIONERS—No. III.

BY SENEX.

As a bilious purgative, applicable in all cases of feverish disturbances, great or small, in their incipiency, there is nothing better than a dose containing ten grains of calomel and half a grain of ipecac. Given at bed-time (a hot foot bath may almost always go with it), it will generally operate on the bowels once or twice itself. It may be given to children and adults alike, and the presence of diarrhœa is no objection.

Discharges, whether sanguineous, serous or mucous, are expressions of disease, not disease themselves; nay, they are generally Nature's *remedies.*

We deal with the status of organs, and with the general system. Ipecac unquestionably gives efficacy to calomel as a purgative, and it certainly does to rhubarb. Dr. Chapman's famous peristaltic persuaders consisted of: ℞—Pulv. rhei ʒj; pulv. ipecac, grs. x; ol. carui, gtt. x. M. ft. pill xx. Two at bedtime.

Bicarb soda, with calomel, is a favorite combination with many judicious physicians.

In the case of a scrofulous boy, with enlarged abdomen, foul tongue, etc., Dr. Pancoast prescribed: ℞—Calomel, gr. i; rhubarb, ij; bicarb soda, v. Every other night.

Dr. Post, of New-York, likes as an alterative, especially for many of the disorders of children: ℞—Pulv. rhei, bicarb soda, aa ʒss; ipecac, grs. iv; aqua anisi ℥iv. A tea-spoonful three times a day. You will find it convenient to prescribe this mixture for all the various cutaneous affections of infancy and childhood, and for any of their anomalous complaints, for which you are sure to be consulted by anxious mothers.

In hot climates, and where there is a tendency to piles, the saline aperients are very comfortable. I had a friend addicted to hemorrhoids, who took a seidlitz powder every morning for thirty years; and Sir B. Brodie tells of an English nobleman who would not go to bed without his aloetic pill. Sir Benjamin did not deny aloes to his hemorrhoidal patients—holding that it was the constipation, and not the drug, which caused the piles. In this view, Dr. Fordyce Barker appears to concur—especially in the hemorrhoids of pregnancy. Dr. Barker is partial to the following: ℞—Aloes, sapo hispan, aa ℈j; ext. hyosciam. ʒ ss; ipecac, grs. v. M. ft. pill No. xx. One twice a day.

Prof. N. R. Smith, of Baltimore, recommends this: ℞—Sulphur loti, ℥j; pulv. senna, ʒj; ol. fœniculi, gtt. xx. Dose, a heaping tea-spoonful every other night.

Fig-paste is very popular among the ladies during gestation. To a pound of figs and an ounce of powdered senna, chopped up and well-mixed together, add a coffee-cup of molasses. Dose, a tea-spoonful or two.

Prunes stewed in senna tea, with the addition of sugar to make a syrup, is also a popular remedy.

The Prussian Pharmacopœia has this, called "Pulv. Glycerina Compound:" ℞—Pulv. sennæ, pulv. glycerina aa ℥ iij; pulv. fennel and sulphur, aa ℥ iss; sacchar. alb., ℥ ix. Dose, a tea-spoonful or more.

Old practitioners retain their preference for castor oil. It is the surest and generally the most thorough of purgatives. A lady, post-partum, said to the nurse, about administering the regular dose of oil on the third day, "Do the doctors give this horrid stuff to all their patients?" and received the very sensible reply, "All prudent ladies take it." Beware of prescribing capsules of castor oil; many of them contain croton oil.

In the constipation of young females, who are apt to neglect their bowels, the late Dr. Bibbins, of New-York, was fond of prescribing: ℞—Quinine, capsici, aa grs. x; aloes, grs. xx; ext. nux vomica, grs. viij. M. ft. pill No. xx. One after dinner and at bed-time. I can testify to the value of the medicine.

Enemeta are of great value. No house should be without a good syringe.

The homœpaths swear by nux vomica as an aperient. A lady friend assures me that she has no trouble in securing regular motions; a tea-spoonful three times a day, of a mixture of ten drops of tinct. of nux vomica in a tumbler of water, never fails. But, then, she has the needed faith, which, we are told on the best authority, can move mountains, not to speak of bowels.

What a blessing has been conferred by Hahnemann on anxious mothers and a drug-loving world! Truly might the surly old moralist exclaim, were he here to see the prevalence of this grand, this truly remarkable humbug: "Medicine is the art of amusing the patient, whilst nature cures the disease." Let us look to it that a truer system come in for no share of the satire, for it was said of us.

Valuable as purgatives are, there are conditions when they are hurtful. In cases of what may be called acute constipation, if the bowels refuse to yield to calomel, castor oil, and injections, better change our tactics. It will be found well to order a hot bath, a full dose of opium, and warm stupes.

We put the bowels in splints in cases of inflammation of the abdominal contents. To illustrate: Robert Carey, aged eight years, was run over by a loaded dray, the wheel passing over his abdomen. He was immediately put on opium, and the bowels locked up for nine days—the case resulting favorably.

Purgatives help the moral hardly less than the physical man. A bilious Englishman visited Voltaire, and discoursing of human life, they pronounced it a failure. The result of the conference was a determination to quit the planet together. The next morning, the punctual Englisher called at Ferney to consummate the plan, poison in hand. "Oh," said the philosopher, "my injection operated '*tres bein*,' and it is a very good world."

Savannah, Georgia, September 14th, 1872.

